# Transcriptional Differences between Rhesus Embryonic Stem Cells Generated from *In Vitro* and *In Vivo* Derived Embryos

**DOI:** 10.1371/journal.pone.0043239

**Published:** 2012-09-18

**Authors:** Alexandra J. Harvey, Shihong Mao, Claudia Lalancette, Stephen A. Krawetz, Carol A. Brenner

**Affiliations:** 1 Department of Physiology, Wayne State University, Detroit, Michigan, United States of America; 2 Center for Molecular Medicine and Genetics, Wayne State University, Detroit, Michigan, United States of America; 3 Department of Pharmacology and Therapeutics, McGill University, Montréal, Québec, Canada; 4 Department of Obstetrics and Gynecology, Wayne State University, Detroit, Michigan, United States of America; 5 Institute for Scientific Computing, Wayne State University, Detroit, Michigan, United States of America; Wellcome Trust Centre for Stem Cell Research, United Kingdom

## Abstract

Numerous studies have focused on the transcriptional signatures that underlie the maintenance of embryonic stem cell (ESC) pluripotency. However, it remains unclear whether ESC retain transcriptional aberrations seen in *in vitro* cultured embryos. Here we report the first global transcriptional profile comparison between ESC generated from either *in vitro* cultured or *in vivo* derived primate embryos by microarray analysis. Genes involved in pluripotency, oxygen regulation and the cell cycle were downregulated in rhesus ESC generated from *in vitro* cultured embryos (*in vitro* ESC). Significantly, several gene differences are similarly downregulated in preimplantation embryos cultured *in vitro*, which have been associated with long term developmental consequences and disease predisposition. This data indicates that prior to derivation, embryo quality may influence the molecular signature of ESC lines, and may differentially impact the physiology of cells prior to or following differentiation.

## Introduction

Embryonic stem cells (ESC) derived from the inner cell mass (ICM) of preimplantation embryos have the potential to differentiate into any cell type of the three embryonic germ layers. ESC retain the ability to proliferate indefinitely, and maintain pluripotency through conserved regulatory networks; however require the provision of various extrinsic factors within the culture environment for continued growth and self-renewal capacity [1], [2]. Loss of pluripotency results in changes in gene expression that include down-regulation of key pluripotency and repressive markers and the up-regulation of regulators of differentiation [3]. Recent studies have documented the transcriptional profiles of various embryonic stem cell lines [4]–[7], establishing a common stem cell regulatory program underlying pluripotency. However, ESC exhibit significant heterogeneity between and within lines, displaying differences in gene expression and differentiation capacity, as well as changes with increasing passage number and culture environment [8]–[11], largely attributed to adaptation with long term culture [12], [13]. Significant differences have also been observed between human ESC lines attributed to differences in derivation techniques [14] and culture conditions [15]–[17]. Very little attention has been paid to other factors which may contribute to the overall normalcy of these cell lines, particularly the quality of the embryo from which a line is derived.

Preimplantation embryo development *in vitro* is associated with a number of perturbations in ultrastructure [18], [19], gene expression [20]–[25] and post-transfer development [26]–[30], when compared with embryos derived *in vivo*. These differences likely underlie the significant variation between ESC lines. There is also considerable evidence that the environment to which the preimplantation embryo is exposed, particularly the *in vitro* culture environment, predisposes the resulting fetus to increased risk of adult onset diseases and imprinting disorders [28], [31]–[36]. Recently, Horii et al [37] reported retention of epigenetic differences in mouse ESC dependent on the *in vivo* or *in vitro* origin of the embryo from which they were derived. While ESC transcriptional profiles are known to differ from that of the ICM [38], [39], these data raise the question as to whether ESC retain transcriptional memory of the embryos from which they were derived. Significantly, it is not clear whether current ESC models are similarly predisposed to developing disease characteristics post-transplantation, or whether they exhibit low levels of perturbation that are not easily distinguishable.

To explore the hypothesis that differences exist between ESC derived from *in vitro* and *in vivo* embryos, gene expression profiles of rhesus macaque ESC generated from either *in vitro* cultured (Ormes series [40]) or *in vivo* derived (R series [41]) embryos were compared.

## Results

### Expression Profiling of rhesus ESC generated from *in vitro* or *in vivo* derived embryos

The transcriptional profiles of undifferentiated ESC generated from either *in vivo* derived or *in vitro* produced rhesus embryos were compared using the Affymetrix GeneChip Rhesus Macaque Genome Array, enabling large scale gene expression profiling of 52,865 probe sets, representing over 20,000 genes. Initial data analysis using dChip software identified a total of 2537 transcripts as significantly different between *in vitro* and *in vivo* ESC, by a twofold or greater fold change (**Table S2**). Comparison between groups revealed 592 probe sets upregulated in rhesus ESC of *in vitro* origin. The reciprocal analysis identified 1945 probe sets upregulated in rhesus ESC of *in vivo* origin. Of the 2537, 1803 had known Entrez Gene IDs. As dChip is a model-based approach that only allows probe-level analysis, we undertook ChipInspector (Genomatix) analysis to assess differences at the level of each gene. ChipInspector identified a total of 3881 transcripts with differential expression of twofold or greater, of which 2706 were unique to the Genomatix analysis (**Table S3**), while 1175 transcripts overlapped with the dChip analysis. Of the 3881 transcripts, 560 genes were upregulated and 3321 were downregulated in *in vitro* ESC.

**Figure 1:**
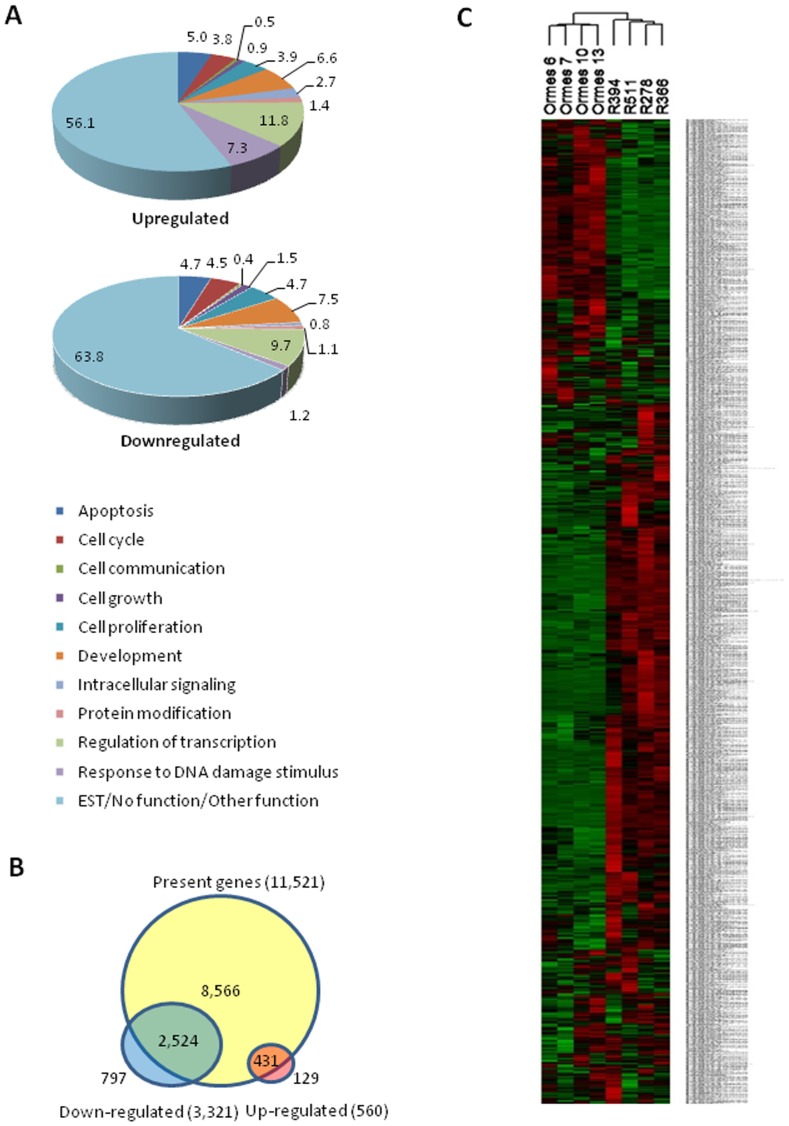
Functional classification and hierarchical clustering of 3881 significantly different transcripts in rhesus ESC. **A:** Pie charts representing up- and down-regulated biological functions of 3881 differentially expression genes in ESC. Numbers represent percentages of 560 up- and 3321 down-regulated genes in ESC generated from *in vitro* cultured embryos, compared with ESC generated from *in vivo* derived embryos. **B:** Combination Venn diagram of shared and specific genes expressed in ESC originating from *in vitro* or *in vivo* derived embryos. The region of overlap between all areas represents the number of genes expressed in ESC from either origin. Regions not overlapping reflect genes expressed specifically in *in vitro* or *in vivo* ESC. There are 11521 genes categorized as present (dChip). Of the 3881 genes identified as significant genes from ChipInspector, 2955 genes are considered as present by dChip, the remaining 926 genes as absent. Of the 2955 genes, 2,524 are down-regulated and 431 are up-regulated; on the 926 absent genes, 797 are down-regulated, 129 are up-regulated. **C:** Dendrogram representing 3881 significantly different transcripts and hierarchical clustering of biological replicates. Colors indicate relative expression level of each gene in all analyzed samples, with red indicating higher expression and green indicating lower expression.

Further classification of the 3881 differentially expressed transcripts by biological function was undertaken using NetAffx (Affymetrix). Several significant (P<0.05) functional biological categories were represented including apoptosis, cell cycle, development and regulation of transcription (**Figure 1A**). Of the 3321 downregulated genes and 560 upregulated genes, 797 and 129 were specific to *in vitro* ESC respectively (**Figure 1B**). Hierarchical clustering demonstrated that gene expression profiles of *in vivo* ESC samples clustered together, separately from *in vitro* ESC samples (**Figure 1C**), indicating that gene expression differences observed between *in vivo* and *in vitro* ESC were greater than differences within the experimental groups.

To identify functional relationships between transcripts, 3881 differentially expressed rhesus transcripts were uploaded into Bibliosphere (Genomatix) for literature based gene connection analysis. Bibliosphere identified 1388 transcripts significantly up- or downregulated in rhesus ESC. Further analysis of the 1388 genes, identified 202 transcription factors (**Table 1**), and 40 significantly enriched pathways (**Table 2**), involving a total of 544 genes.

**Table 1:** Transcription factor expression significantly altered by ESC origin.

Gene symbol	Gene name	q-value
PAX8	paired box 8	2.16
NR6A1	nuclear receptor subfamily 6, group A, member 1	2.07
HIVEP3	human immunodeficiency virus type I enhancer binding protein 3	2.02
TAF1	TBP-associated factor 1	1.82
NFATC1	nuclear factor of activated T-cells, cytoplasmic, calcineurin-dependent 1	1.68
ZNF219	zinc finger protein 219	1.62
ARID2	AT rich interactive domain 2 (ARID, RFX-like)	1.617
SHOX2	short stature homeobox 2	1.56
ETV5	ets variant 5	1.56
FOXJ3	forkhead box J3	1.55
SMAD2	SMAD family member 2	1.5
ZNF292	zinc finger protein 292	1.5
RBPJ	recombination signal binding protein for immunoglobulin kappa J region	1.49
E2F7	E2F transcription factor 7	1.46
ZFX	zinc finger protein, X-linked	1.45
ZNF280B	zinc finger protein 280B	1.39
KLF3	Kruppel-like factor 3 (basic)	1.36
BAZ2B	bromodomain adjacent to zinc finger domain, 2B	1.36
ZNF24	zinc finger protein 24	1.36
TBP	TATA box binding protein	1.34
UBN1	ubinuclein 1	1.31
RFX7	regulatory factor X, 7	1.26
TIAM1	T-cell lymphoma invasion and metastasis 1	1.25
MTF2	metal response element binding transcription factor 2	1.242
SLC30A9	solute carrier family 30 (zinc transporter), member 9	1.11
SETDB1	SET domain, bifurcated 1	1.1
CDCA7	cell division cycle associated 7	1.01
ZNF148	zinc finger protein 148	0.41
GTF2H2	general transcription factor IIH, polypeptide 2, 44 kDa	0.27
NCOA3	nuclear receptor coactivator 3	0.259
PYGO2	pygopus homolog 2 (Drosophila)	0.055
RBM4	RNA binding motif protein 4	0.02
CDK8	cyclin-dependent kinase 8	0.005
ATRX	alpha thalassemia/mental retardation syndrome X-linked (RAD54 homolog, S. cerevisiae)	−0.14
PUF60	poly-U binding splicing factor 60 KDa	−0.175
SP3	Sp3 transcription factor	−0.297
NPAT	nuclear protein, ataxia-telangiectasia locus	−0.56
SMARCA1	SWI/SNF related, matrix associated, actin dependent regulator of chromatin, subfamily a, member 1	−0.586
SMAD3	SMAD family member 3	−0.629
ASH2L	ash2 (absent, small, or homeotic)-like	−0.923
ZMYM2	zinc finger, MYM-type 2	−0.94
IRF3	interferon regulatory factor 3	−1.01
MED12	mediator complex subunit 12	−1.01
ZNF215	zinc finger protein 215	−1.01
HIPK3	homeodomain interacting protein kinase 3	−1.02
TAF6L	TAF6-like RNA polymerase II	−1.02
PHF19	PHD finger protein 19	−1.02
ING1	inhibitor of growth family, member 1	−1.02
MLL	myeloid/lymphoid or mixed-lineage leukemia (trithorax homolog, Drosophila)	−1.03
ZNF192	zinc finger protein 192	−1.03
NCOA2	nuclear receptor coactivator 2	−1.04
TP53	tumor protein p53	−1.04
MEF2A	myocyte enhancer factor 2A	−1.04
SATB1	SATB homeobox 1	−1.04
PHTF2	putative homeodomain transcription factor 2	−1.046
HOXB1	homeobox B1	−1.05
ZNF76	zinc finger protein 76 (expressed in testis)	−1.05
MED1	mediator complex subunit 1	−1.05
MYBL1	v-myb myeloblastosis viral oncogene homolog (avian)-like 1	−1.05
TRIP11	thyroid hormone receptor interactor 11	−1.05
HSF1	heat shock transcription factor 1	−1.05
MYCN	v-myc myelocytomatosis viral related oncogene, neuroblastoma derived (avian)	−1.06
ZEB1	zinc finger E-box binding homeobox 1	−1.06
MAML2	mastermind-like 2 (Drosophila)	−1.06
MYST1	MYST histone acetyltransferase 1	−1.06
SCML1	sex comb on midleg-like 1 (Drosophila)	−1.06
TLE4	transducin-like enhancer of split 4 (E(sp1) homolog, Drosophila)	−1.065
CNOT3	CCR4-NOT transcription complex, subunit 3	−1.07
SP1	Sp1 transcription factor	−1.07
DEAF1	deformed epidermal autoregulatory factor 1	−1.08
TARBP2	TAR (HIV-1) RNA binding protein 2	−1.08
SIX4	SIX homeobox 4	−1.08
CDK9	cyclin-dependent kinase 9	−1.08
CREBL2	cAMP responsive element binding protein-like 2	−1.08
TRIM33	tripartite motif-containing 33	−1.09
RNF14	ring finger protein 14	−1.09
PRIC285	PPAR-alpha interacting complex protein 285	−1.1
TMF1	TATA element modulatory factor 1	−1.1
PURA	similar to Transcriptional activator protein Pur-alpha (Purine-rich single-stranded DNA-binding protein alpha)	−1.1
NCOR2	nuclear receptor co-repressor 2	−1.102
YAF2	YY1 associated factor 2	−1.103
HESX1	HESX homeobox 1	−1.12
ELF2	similar to E74-like factor 2 (ets domain transcription factor) isoform 2	−1.12
FOXN3	forkhead box N3	−1.13
HSF2	heat shock transcription factor 2	−1.14
ZFP36L2	zinc finger protein 36, C3H type-like 2	−1.14
ACTR5	ARP5 actin-related protein 5 homolog (yeast)	−1.15
SMAD4	SMAD family member 4	−1.17
DDX54	DEAD (Asp-Glu-Ala-Asp) box polypeptide 54	−1.17
POU5F1	POU class 5 homeobox 1	−1.17
ZSCAN21	zinc finger and SCAN domain containing 21	−1.176
ERCC3	excision repair cross-complementing rodent repair deficiency, complementation group 3	−1.18
STAT1	signal transducer and activator of transcription 1	−1.185
ZNF81	zinc finger protein 81	−1.2
HMGA2	high mobility group AT-hook 2	−1.205
INGX	inhibitor of growth family, X-linked, pseudogene	−1.21
ZNF140	zinc finger protein 140	−1.21
DIDO1	death inducer-obliterator 1	−1.22
ARNTL	aryl hydrocarbon receptor nuclear translocator-like	−1.226
NAB2	NGFI-A binding protein 2	−1.228
BAZ1A	bromodomain adjacent to zinc finger domain, 1A	−1.23
SSBP1	single-stranded DNA binding protein 1	−1.23
CREG1	cellular repressor of E1A-stimulated genes 1	−1.24
HCFC1	host cell factor C1 (VP16-accessory protein)	−1.25
MYBBP1A	MYB binding protein (P160) 1a	−1.25
MLX	MAX-like protein X	−1.262
KLF5	similar to Krueppel-like factor 5	−1.28
TAF2	TAF2 RNA polymerase II, TATA box binding protein (TBP)-associated factor, 150 kDa	−1.285
PIAS2	protein inhibitor of activated STAT, 2	−1.285
PHF10	PHD finger protein 10	−1.29
SMAD1	SMAD family member 1	−1.297
ELL2	elongation factor, RNA polymerase II, 2	−1.31
ETV6	ets variant 6	−1.313
ETS1	v-ets erythroblastosis virus E26 oncogene homolog 1 (avian)	−1.317
TP53BP2	tumor protein p53 binding protein, 2	−1.33
ZNF143	zinc finger protein 143	−1.33
MED7	mediator complex subunit 7	−1.33
BTF3	basic transcription factor 3	−1.34
ZNF410	zinc finger protein 410	−1.34
FOXO1	forkhead box O1	−1.34
STAT3	signal transducer and activator of transcription	−1.345
DR1	down-regulator of transcription 1, TBP-binding (negative cofactor 2)	−1.35
CTCF	similar to Transcriptional repressor CTCF (CCCTC-binding factor) (CTCFL paralog) (11-zinc finger protein)	−1.35
GTF2H4	general transcription factor IIH, polypeptide 4, 52 kDa	−1.35
SAP18	Sin3A-associated protein, 18 kDa	−1.35
ACTL6A	actin-like 6A	−1.36
TFDP2	transcription factor Dp-2 (E2F dimerization partner 2)	−1.366
CNOT2	CCR4-NOT transcription complex, subunit 2	−1.37
BHLHE40	basic helix-loop-helix family, member e40	−1.38
KDM3A	lysine (K)-specific demethylase 3A	−1.38
BRD7	bromodomain containing 7	−1.38
GTF2F1	general transcription factor IIF, polypeptide 1, 74 kDa	−1.39
BCOR	BCL6 co-repressor	−1.39
ZNF281	zinc finger protein 281	−1.39
TFAP2C	transcription factor AP-2 gamma	−1.39
SAP30	Sin3A-associated protein, 30 kDa	−1.4
MED17	mediator complex subunit 17	−1.4
ZNF451	zinc finger protein 451	−1.42
TCF7L2	transcription factor 7-like 2 (T-cell specific, HMG-box)	−1.44
SMAD5	SMAD family member 5	−1.44
RB1	retinoblastoma 1	−1.45
JMJD1C	jumonji domain containing 1C	−1.451
ATF1	activating transcription factor 1	−1.47
CREB1	cAMP responsive element binding protein 1	−1.48
THRAP3	thyroid hormone receptor associated protein 3	−1.49
YBX1	Y box binding protein 1	−1.5
GTF2H1	general transcription factor IIH, polypeptide 1, 62 kDa	−1.508
MECP2	methyl CpG binding protein 2 (Rett syndrome)	−1.51
TAF12	TAF12 RNA polymerase II, TATA box binding protein (TBP)-associated factor, 20 kDa	−1.51
CBFB	core-binding factor, beta subunit	−1.52
MED20	mediator complex subunit 20	−1.52
DDX20	DEAD (Asp-Glu-Ala-Asp) box polypeptide 20	−1.53
WDR77	WD repeat domain 77	−1.545
BTAF1	BTAF1 RNA polymerase II, B-TFIID transcription factor-associated, 170 kDa	−1.55
TAF9	TAF9 RNA polymerase II, TATA box binding protein (TBP)-associated factor, 32 kDa	−1.56
MED19	mediator complex subunit 19	−1.578
PIAS1	protein inhibitor of activated STAT, 1	−1.587
CNOT8	CCR4-NOT transcription complex, subunit 8	−1.59
NRIP1	nuclear receptor interacting protein 1	−1.61
TSG101	tumor susceptibility gene 101	−1.62
MED10	mediator complex subunit 10	−1.62
KAT5	K(lysine) acetyltransferase 5	−1.63
SMARCA4	SWI/SNF-related matrix-associated actin-dependent regulator of chromatin a4	−1.65
ABT1	activator of basal transcription 1	−1.67
SMARCC1	SWI/SNF-related matrix-associated actin-dependent regulator of chromatin c1	−1.67
ETS2	v-ets erythroblastosis virus E26 oncogene homolog 2	−1.68
ZNF462	zinc finger protein 462	−1.7
SOX2	SRY (sex determining region Y)-box 2	−1.71
ZNF423	zinc finger protein 423	−1.72
CTNNB1	catenin (cadherin-associated protein), beta 1, 88 kDa	−1.76
FUBP1	far upstream element (FUSE) binding protein 1	−1.77
HBP1	HMG-box transcription factor 1	−1.78
CREM	cAMP responsive element modulator	−1.8
TFAM	transcription factor A, mitochondrial	−1.8
PTTG1	pituitary tumor-transforming 1	−1.81
CCND1	cyclin D1	−1.81
ATF4	activating transcription factor 4 (tax-responsive enhancer element B67)	−1.83
TRRAP	transformation/transcription domain-associated protein	−1.885
HIVEP1	human immunodeficiency virus type I enhancer binding protein 1	−1.9
CALR	calreticulin	−1.92
ADNP	activity-dependent neuroprotector homeobox	−1.93
MYC	v-myc myelocytomatosis viral oncogene homolog (avian)	−1.94
TCEA1	transcription elongation factor A (SII), 1	−2.01
CITED2	similar to Cbp/p300-interacting transactivator, with Glu/Asp-rich carboxy-terminal	−2.06
ID4	inhibitor of DNA binding 4, dominant negative helix-loop-helix protein	−2.075
TCEB3	transcription elongation factor B (SIII), polypeptide 3 (110 kDa, elongin A)	−2.08
YWHAH	tyrosine 3-monooxygenase/tryptophan 5-monooxygenase activation protein, eta polypeptide	−2.12
DDX5	DEAD (Asp-Glu-Ala-Asp) box polypeptide 5	−2.13
ANKRD1	ankyrin repeat domain 1 (cardiac muscle)	−2.18
GTF3A	general transcription factor IIIA	−2.27
COPS5	COP9 constitutive photomorphogenic homolog subunit 5 (Arabidopsis)	−2.295
HTATSF1	HIV-1 Tat specific factor 1	−2.3
NFYB	nuclear transcription factor Y, beta	−2.342
STRAP	serine/threonine kinase receptor associated protein	−2.457
HIF1A	hypoxia inducible factor 1, alpha subunit (basic helix-loop-helix transcription factor)	−2.462
BCLAF1	BCL2-associated transcription factor 1	−2.49
GTF2I	general transcription factor II	−2.56
MORF4L2	similar to Mortality factor 4-like protein 2 (MORF-related gene X protein) (Transcription factor-like protein MRGX) (MSL3-2 protein)	−2.8
PFN1	profilin 1	−2.82
TARDBP	TAR DNA binding protein	−2.89
DDX17	DEAD (Asp-Glu-Ala-Asp) box polypeptide 17	−2.96
HELLS	helicase, lymphoid-specific	−2.965

Higher ratios represent genes upregulated in in vitro ESC, lower ratios are upregulated in in vivo ESC. As ChipInspector considers one probe as significant if the fold-change is greater than 2, the final FC for each gene represents the average of all probes that overlap the gene. The q-value is calculated as log2 fold change.

**Table 2:** Canonical signal transduction pathways represented by the 1388 differentially expressed transcripts from ESC generated from either *in vivo* derived or *in vitro* cultured embryos.

Canonical pathway	P-value	# Genes (observed)	# Genes (expected)	Total genes in pathway	List of observed genes
Androgen Receptor	1.01E-06	28	10.94444	87	STUB1, CTNNB1, AKT1, HIPK3, CALR, PXN, SVIL, MAPK1, STAT3, SP1, TMF1, NCOA3, CDK9, CDC37, CDC2, RB1, MDM2, SMAD3, PIAS1, RNF14, CCNH, NCOR2, GTF2F1, PTEN, NCOA2, CAV1, NRIP1, GTF2H4
HIV-1 NEF: negative effector of FAS and TNF	1.4E-05	19	6.793103	54	NUMA1, LMNB1, PSEN1, CASP8, GSN, LMNA, MAP3K1, BIRC2, RB1, PAK2, MDM2, CFLAR, RASA1, FAS, CHUK, PTK2, CASP3, PSEN2, BAG4
Osteopontin-mediated events	0.000137	12	3.773946	30	PIK3R1, MMP2, VAV3, GSN, SPP1, MAPK1, MAP3K1, CD44, ROCK2, CHUK, PLAU, MAPK3
Integrins in angiogenesis	0.000243	16	6.289911	50	PIK3R1, VEGFA, AKT1, CASP8, VAV3, PXN, TLN1, SPP1, MAPK1, FGF2, SDC1, IGF1R, HSP90AA1, PI4KB, PTK2, MAPK3
VEGFR1 specific signals	0.000315	11	3.52235	28	PLCG1, PIK3R1, VEGFA, AKT1, NRP2, HIF1A, MAPK1, HSP90AA1, RASA1, CAV1, MAPK3
FAS signaling pathway (cd95)	0.000338	9	2.515964	20	CASP8, MAP3K1, FAF1, RB1, PAK2, CFLAR, FAS, MAP3K7, CASP3
Mechanism of gene regulation by peroxisome proliferators via ppara	0.00037	14	5.283525	42	DUSP1, MYC, CITED2, MED1, MAPK1, SP1, DUT, RB1, HSD17B4, HSP90AA1, ME1, NCOR2, NRIP1, MAPK3
Rb tumor suppressor/checkpoint signaling in response to dna damage	0.000411	7	1.635377	13	YWHAH, CDK4, TP53, WEE1, CDC2, RB1, CDK2
HIF-1-alpha transcription factor network	0.000469	19	8.554278	68	VEGFA, AKT1, HIF1A, CITED2, SP1, MCL1, HMOX1, BHLHE40, ETS1, PGK1, SMAD3, TFRC, CREB1, NCOA2, EDN1, ADM, COPS5, CXCL12
Human cytomegalovirus and map kinase pathways	0.000505	8	2.13857	17	PIK3R1, AKT1, MAPK1, SP1, MAP3K1, RB1, CREB1, MAPK3
TGFBR	0.000593	32	17.98914	143	SNX1, SMAD2, PIK3R1, CTNNB1, CDK4, TP53, STRAP, CUL1, SNX4, MYC, NFYB, UBE2D1, CAMK2D, SP1, TGFB1, CDK6, TFDP2, CDC16, ETS1, CDC2, CTCF, RB1, SMAD3, CD44, CAMK2G, SNX2, PIAS1, CDK2, MAP3K7, CAV1, MEF2A, COPS5
Angiopoietin receptor Tie2-mediated signaling	0.000648	15	6.164112	49	PLG, PIK3R1, FOXO1, AKT1, ITGA5, MMP2, PXN, MAPK1, ELF2, FGF2, ETS1, RASA1, FYN, PTK2, MAPK3
FAS signaling pathway (CD95)	0.000729	12	4.402937	35	CASP8, GSN, LMNA, MAP3K1, FAF1, RB1, PAK2, CFLAR, FAS, CHUK, MAP3K7, CASP3
Co-regulation of Androgen receptor activity	0.000779	17	7.547893	60	CTNNB1, CTDSP2, AKT1, XRCC5, CASP8, MED1, VAV3, SVIL, GSN, CDK6, TMF1, TCF4, PIAS1, FKBP4, KDM3A, NCOA2, NRIP1
EGF receptor proximal signaling	0.001023	10	3.396552	27	PLCG1, PTPN1, GSN, WASL, MAPK1, STAT3, GNAI3, RASA1, PTK2, MAPK3
Estrogen responsive protein eEFP controls cell cycle and breast tumors growth	0.001229	7	1.886973	15	CDK4, TP53, CDK8, CDK6, CDC2, CCNB1, CDK2
Cell cycle: G1/S check point	0.001415	10	3.52235	28	CDK4, TP53, SKP2, TGFB1, CDK6, TK1, CDC2, RB1, SMAD3, CDK2
Transcription factor CREBb and its extracellular signals	0.001415	10	3.52235	28	PRKAR2B, PIK3R1, AKT1, CAMK2D, PRKAR1A, MAPK1, ASAH1, CAMK2G, CREB1, MAPK3
NOTCH	0.002404	19	9.686462	77	SMAD1, HIVEP3, PIK3R1, JAG1, SKP2, MAML2, RBPJ, ADAM10, CUL1, PSEN1, SAP30, MAPK1, STAT3, APP, FHL1, SMAD3, NCOR2, PSEN2, MAPK3
Migration	0.002424	36	22.64368	180	PRKAR2B, PLCG1, MAPKAPK3, PIK3R1, CDK4, VEGFA, AKT1, ZAP70, CAMK2D, PRKAR1A, RYK, PRKCI, MAPK1, CDK8, WEE1, CDK6, MAP3K12, CDK9, ITPR1, MAP3K1, CDC2, IGF1R, PAK2, MAPKAPK2, CSNK1A1, CAMK2G, PIK3CB, AKT2, CDK2, CHUK, CCNH, FYN, MAP3K7, PTK2, NGFR, MAPK3
Signaling events mediated by VEGFR1 and VEGFR2	0.002466	17	8.302682	66	PLCG1, HSPB1, PIK3R1, CTNNB1, VEGFA, AKT1, NRP2, HIF1A, PXN, MAPK1, HSP90AA1, IQGAP1, FYN, GRB10, PTK2, CAV1, MAPK3
E-cadherin signaling in keratinocytes	0.002676	8	2.641762	21	PLCG1, PIK3R1, CTNNB1, AKT1, CTNNA1, CTNND1, AKT2, FYN
Regulation of glucocorticoid receptor	0.002693	11	4.402937	35	YWHAH, TP53, AKT1, SMARCC1, SMARCA4, MAPK1, MDM2, HSP90AA1, FKBP4, NCOA2, MAPK3
Platelet amyloid precursor protein pathway	0.003007	6	1.635377	13	PLG, COL4A6, PLAT, COL4A5, APP, PLAU
p53 signaling pathway	0.003007	6	1.635377	13	CDK4, TP53, TIMP3, RB1, MDM2, CDK2
FOXM1 transcription factor network	0.004236	12	5.283525	42	CDK4, SKP2, MYC, MMP2, CENPA, SP1, NEK2, CDC2, RB1, CCNB1, AURKB, CDK2
ERK and PI-3 kinase necessary for collagen binding in corneal epithelia	0.004374	10	4.025543	32	PLCG1, PIK3R1, PXN, GSN, TLN1, MAPK1, PFN1, PTK2, DIAPH1, MAPK3
TNF alpha/NF-kB	0.004456	33	21.0083	167	HSPB1, POLR2L, YWHAH, AKT1, CUL1, ALPL, TRAF6, CASP8, CASP8AP2, SMARCC1, SMARCA4, KPNA3, TNIP1, MCM5, MAP3K1, BCL7A, LRPPRC, FAF1, BIRC2, CDC37, KPNA6, PSMD3, HSP90AA1, AKT2, CFLAR, COPS3, CHUK, CASP3, CAV1, ACTL6A, BAG4, AZI2, MAP3K7IP2
How progesterone initiates oocyte maturation	0.005132	8	2.893359	23	PRKAR2B, PRKAR1A, CAP1, CDC25C, MAPK1, CDC2, CCNB1, MAPK3
Cyclins and cell cycle regulation	0.005132	8	2.893359	23	CDK4, CCND2, CDK6, CDC2, RB1, CCNB1, CDK2, CCNH
CTCF: first multivalent nuclear factor	0.005132	8	2.893359	23	SMAD1, PIK3R1, MYC, TGFB1, CTCF, MDM2, SMAD5, PTEN
IFN-gamma pathway	0.00523	12	5.409323	43	PIK3R1, AKT1, DAPK1, CAMK2D, MAPK1, STAT3, MAP3K1, IFNGR1, CAMK2G, PIAS1, CRKL, MAPK3
Akt signaling pathway	0.006137	7	2.390166	19	GHR, PIK3R1, YWHAH, FOXO1, AKT1, HSP90AA1, CHUK
Overview of telomerase RNA component gene hTERC transcriptional regulation	0.006296	4	0.880587	7	NFYB, SP1, SP3, RB1
AKT(PKB)-Bad signaling	0.006818	34	22.39208	178	PRKAR2B, MAPKAPK3, PIK3R1, CDK4, AKT1, ZAP70, CAMK2D, PRKAR1A, RYK, PRKCI, MAPK1, STAT3, CDK8, WEE1, CDK6, MAP3K12, CDK9, MAP3K1, CDC2, IGF1R, PAK2, MAPKAPK2, CSNK1A1, CAMK2G, PIK3CB, AKT2, CDK2, CHUK, CCNH, FYN, MAP3K7, PTK2, NGFR, MAPK3
Generation of amyloid b-peptide by ps1	0.006922	3	0.503193	4	ADAM10, PSEN1, APP
Influence of ras and rho proteins on g1 to s transition	0.007125	9	3.648148	29	PIK3R1, CDK4, AKT1, MAPK1, CDK6, RB1, CDK2, CHUK, MAPK3
p75(NTR)-mediated signaling	0.007285	16	8.42848	67	PLG, PIK3R1, TP53, AKT1, PSEN1, BCL2L11, TRAF6, PRKCI, APP, BIRC2, CHUK, RTN4, CASP3, NGFR, ARHGDIA, SORT1
VEGF hypoxia and angiogenesis	0.009077	9	3.773946	30	PLCG1, PIK3R1, VEGFA, AKT1, HIF1A, PXN, HSP90AA1, PTK2, CAV1
TNF receptor signaling pathway	0.009336	12	5.786718	46	MAP4K5, CASP8, PRKCI, SMPD1, MAP3K1, BIRC2, CHUK, MAP3K7, CAV1, BAG4, MAP3K7IP2, TNIK

**Figure 2:**
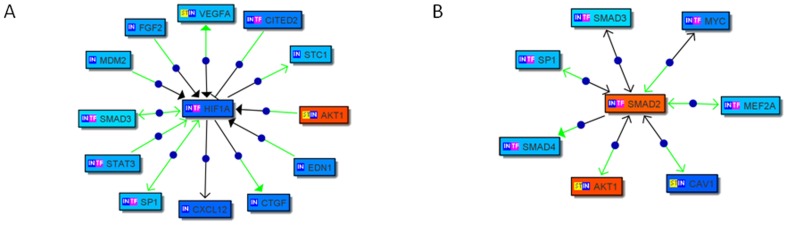
Bibliosphere analysis of transcripts where two genes are co-cited and restricted to sentences with gene+function word+gene. sentences with expert curated information. Each rectangle depicts a single gene. Red indicates the gene is unregulated, blue downregulated. Arrows between two genes shows regulatory mechanisms: green indicates a transcription factor binding site match in the target promoter; open arrowhead indicates regulation; filled arrowhead indicates activation; blocked arrowhead indicates inhibition; blue dot on the edge indicates that the connection has been annotated by experts; **A:** Associations present between HIF1A and other genes at the expert level; **B:** Associations present between SMAD2 and other genes at the expert level. IN: gene is an input gene; TF: gene's product is a transcription factor; ST: gene product is part of signal transduction pathway.

Of the 202 transcription factors identified in Bibliosphere four known to be involved in the transcriptional control of pluripotency, POU5F1, Akt, SMAD2 and HIF1A, were further analyzed to establish literature based gene networks. The interactions of HIF1A and SMAD2 with other genes are presented in **Figure 2**. Regulatory mechanisms of the transcription factors HIF1A (Matrix family HIFF) and SMAD2 (Matrix family SMAD)'s were further studied as shown in **Figure 2**. The promoter regions of eleven genes were found to have HIFF binding sites. Likewise, the promoter regions of five genes contained SMAD binding sites.

Common framework, a pattern of transcription factor binding sites defined by a set of physical parameters such as order, distance, and strand orientation on the promoter region, is a promoter module that participates in transcription regulation in a certain context. The common frameworks were mined from the eleven genes' and five genes' promoter regions identified above. Frameworks CTCF-HIFF, ETSF-HIFF and SMAD-E2FF were identified in these two gene groups respectively and suggest that transcription factors CTCF and ETSF may work with HIFF, and that E2FF may work with SMAD, to regulate transcription (**Table S4**).

### Expression of markers of pluripotency

Comparison of the 1388 significant differentially expressed genes with previous microarray data examining regulators of pluripotency [4]–[6], [16], [42]–[47] identified 225 significantly different genes documented by at least one publication, with 68 of these genes documented by at least two or more publications (**Table 3**). Among these genes *FGF2* (basic FGF) and *FGFR1* were significantly downregulated (2-fold) in *in vitro* ESC. Similarly, *SOX2* expression was decreased more than 3-fold in *in vitro* ESC, while *POU5F1* was reduced by 2-fold. Other genes, including those involved in transcriptional repression and TGFß signaling, were also identified. In particular *TGFß1*, *FST*, *SMAD1*, *4* and *5* and *ID4* were downregulated in *in vitro* ES, while *SMAD3* was upregulated (**Table S3**).

**Table 3:** Altered expression pattern of known markers of pluripotency.

Gene Symbol	Gene Name	q-value	References
ADSL	adenylosuccinate lyase	−1.56	[4], [6], [47]
ALDH3A2	aldehyde dehydrogenase 3 family, member A2	−1.402	[6], [45]
ALPL	alkaline phosphatase, liver/bone/kidney	−1.25	[6], [47]
ASPM	asp (abnormal spindle) homolog, microcephaly associated (Drosophila)	−1.1	[16], [45]
BST2	bone marrow stromal cell antigen 2	−2.215	[16], [45]
CBR1	carbonyl reductase 1	−1.3	[6], [45]
CCNB1	cyclin B1	1.582	[4], [6], [47]
CCNC	cyclin C	−1.43	[4], [44], [47]
CCND1	cyclin D1	−1.81	[6], [44], [45]
CCNF	cyclin F	2.17	[16], [44]
CDC2	cell division cycle 2, G1 to S and G2 to M	−1.773	[4], [6], [43], [44], [47]
CDKN3	cyclin-dependent kinase inhibitor 3	−1.1	[6], [45]
COMMD3	COMM domain containing 3	−1.2	[5], [42]
CRABP1	cellular retinoic acid binding protein 1	−2.43	[4], [6], [44], [47]
CTSC	cathepsin C	−2.135	[6], [45]
CUL1	cullin 1	−1.775	[16], [44]
DKC1	dyskeratosis congenita 1, dyskerin	−0.09	[6], [47]
DSG2	desmoglein 2	−1.87	[4], [47]
ECT2	epithelial cell transforming sequence 2 oncogene	−1.82	[6], [43]
EEF1B2	eukaryotic translation elongation factor 1 beta 2	−1.35	[6], [47]
EPRS	glutamyl-prolyl-tRNA synthetase	−1.71	[4], [43], [47]
FABP5	fatty acid binding protein 5 (psoriasis-associated)	−2.28	[4], [6], [47]
FGF2	fibroblast growth factor 2 (basic)	−1.465	[5], [6], [45]
FGFR1	fibroblast growth factor receptor 1	−1.024	[5], [6]
FKBP4	FK506 binding protein 4, 59 kDa	−1.26	[6], [44]
GABRB3	gamma-aminobutyric acid (GABA) A receptor, beta 3	−1.643	[16], [42], [45]
GART	phosphoribosylglycinamide formyltransferase, phosphoribosylglycinamide synthetase, phosphoribosylaminoimidazole synthetase	−1.5	[6], [16], [47]
GPC4	glypican 4	−2.04	[6], [43], [47]
GPM6B	glycoprotein M6B	−1.03	[6], [45]
HELLS	helicase, lymphoid-specific	−2.965	[6], [16], [44], [45]
HNRNPA2B1	heterogeneous nuclear ribonucleoprotein A2/B1	−3.238	[6], [44]
HNRNPAB	heterogeneous nuclear ribonucleoprotein A/B	−2.91	[43], [47]
IDH1	isocitrate dehydrogenase 1 (NADP+), soluble	−2.32	[4], [6], [47]
IMPDH2	IMP (inosine monophosphate) dehydrogenase 2	−1.85	[4], [47]
KIF5C	kinesin family member 5C	−1.25	[6], [45]
LTA4H	leukotriene A4 hydrolase	−1.46	[6], [45]
MAD2L2	MAD2 mitotic arrest deficient-like 2 (yeast)	−1.52	[4], [47]
MCM7	minichromosome maintenance complex component 7	−1.705	[6], [47]
MGST1	microsomal glutathione S-transferase 1	−2.38	[4], [47]
MKRN1	makorin ring finger protein 1	1.38	[6], [44]
MPHOSPH9	M-phase phosphoprotein 9	−1.15	[6], [16]
MSH2	mutS homolog 2	−1.94	[6], [44], [46]
NEK2	NIMA (never in mitosis gene a)-related kinase 2	−1.822	[6], [44]
NFYB	nuclear transcription factor Y, beta	−2.342	[6], [44], [45]
PGK1	phosphoglycerate kinase 1	−1.462	[6], [47]
PIM1	pim-1 oncogene	1.63	[6], [47]
POU5F1	POU class 5 homeobox 1	−1.17	[4]–[6], [16], [44]–[47]
PPAT	phosphoribosyl pyrophosphate amidotransferase	−1.345	[4], [6], [43], [45], [47]
PSMA2	proteasome (prosome, macropain) subunit, alpha type, 2	−2.03	[4], [6], [44], [47]
PSMD14	proteasome (prosome, macropain) 26S subunit, non-ATPase, 14	−1.42	[46], [47]
PTPRZ1	protein tyrosine phosphatase, receptor-type, Z polypeptide 1	−2.602	[4], [6], [45]
PTTG1	pituitary tumor-transforming 1	−1.81	[6], [47]
SCG3	secretogranin III	−1.115	[7], [16]
SERPINH1	serpin peptidase inhibitor, clade H (heat shock protein 47), member 1, (collagen binding protein 1)	−4.02	[4], [47]
SLC16A1	solute carrier family 16, member 1	−2.693	[4], [6], [47]
SLC29A1	solute carrier family 29 (nucleoside transporters), member 1	−1.53	[6], [45]
SNRPA1	small nuclear ribonucleoprotein polypeptide A′	−1.52	[6], [47]
SNX5	sorting nexin 5	−1.416	[6], [16]
SOD1	superoxide dismutase 1, soluble	−1.57	[6], [44]
SOX2	SRY (sex determining region Y)-box 2	−1.71	[5], [45]
TCEA1	transcription elongation factor A (SII), 1	−2.01	[43], [46]
TFAP2C	transcription factor AP-2 gamma	−1.39	[5], [43], [44]
THY1	Thy-1 cell surface antigen	−1.815	[6], [45]
TK1	thymidine kinase 1, soluble	−1.2	[4], [43], [47]
TKT	similar to Transketolase (TK)	−1.947	[6], [43], [47]
UGP2	UDP-glucose pyrophosphorylase 2	−1.25	[6], [16], [43], [47]
USP9X	ubiquitin specific peptidase 9, X-linked	−2.178	[6], [43], [44]
XRCC5	X-ray repair complementing defective repair in Chinese hamster cells 5 (double-strand-break rejoining)	−2.527	[6], [47]

Comparison of results of differentially expressed genes between rhesus ESC generated from in vitro or in vivo derived embryos, with previously documented microarray results of human ESC, identified 68 genes reported by at least two publications as markers of pluripotency. The q-value is calculated as log2 fold change.

### Differentially expressed genes correlate with differences observed in preimplantation embryos

Analysis was undertaken to determine whether ESC generated from *in vitro* cultured rhesus embryos displayed perturbations in gene expression reported in the literature as differentially expressed in *in vitro* and *in vivo* preimplantation embryos [19], [23], [26], [28], [31], [48]–[52], results of which are summarized in **Table 4**. These differences included significantly decreased expression of insulin-like growth factor receptor 1 and 2 (*IGF-I*, *IGF-II*), glucose transporters 3 and 5 (*SLC2A3*, *SLCA2A5*), activating transcription factor 1 (*ATF1*), cyclin D1, secreted phosphoprotein 1, and the antioxidant enzymes superoxide dismutase 1 (*SOD1*), peroxiredoxin 2 (*PDX2*) and glutathione peroxidase 4 (*GPX4*) was seen in *in vitro* ESC. Alterations in gene expression observed in mouse embryos as a result of the use of serum during embryo culture [52] were also detected, and included downregulation of platelet derived growth factor receptor (*PDGFR*), the metabolic genes pyruvate dehydrogenase isoenxyme 1, aldehyde dehydrogenase 2 (*ADH2*) and aldehyde dehydrogenase family 6 subfamily A1, and upregulation of solute carrier family 25 (mitochondrial carrier, citrate transporter) member 1.

**Table 4:** Differentially expressed transcripts that display altered expression patterns following *in vitro* embryo culture.

Gene ID	Gene Symbol	Gene Name	UnigeneID	Gene Bank Accession	q-value
693644	ATF1	activating transcription factor 1	Mmu.12123	XM_001083228	−1.47
713451	ALDH2	mitochondrial aldehyde	Mmu.9621	XR_012809	−2.25
		dehydrogenase 2		AANU01210495	
				AANU01210500	
				AANU01210496	
				AANU01210497	
				AANU01210498	
				AANU01210499	
698755	ALDH6A1	aldehyde dehydrogenase 6	Mmu.11793	XM_001093055	−1.50
		family, member A1		XM_001093276	
717809	ALPL	alkaline phosphatase, liver/bone/kidney	#N/A	XM_001109717	−1.25
574320	CCND1	cyclin D1	Mmu.3863	AY950561	−1.81
				XM_001101029	
707479	F2RL1	coagulation factor II	#N/A	XM_001106201	−2.78
		(thrombin) receptor-like 1		XM_001106263	
574136	FGF2	fibroblast growth factor 2	Mmu.3766	XM_001099284	−1.47
		(basic)		AF251270	
697986	GHR	growth hormone receptor	Mmu.3595	XM_001088963	−1.16
				XM_001088858	
				U85396	
				U84589	
				NM_001042667	
705333	GPX4	glutathione peroxidase 4	Mmu.9752	AANU01110880	−2.07
				CB552751	
				NM_001118889	
				CN643832	
				XR_011424	
697821	HEBP1	heme binding protein 1	Mmu.11875	XM_001086941	−1.29
708227	IGF1R	insulin-like growth factor 1 receptor	#N/A	XM_001100407	−1.07
703220	IGF2R	insulin-like growth factor	Mmu.7995	XR_012149	−1.11
		2 receptor		AANU01296649	
				AANU01296648	
				AANU01296647	
				AANU01296646	
				AANU01296645	
				AANU01296643	
				AANU01296644	
				AANU01296641	
				AANU01296642	
				AANU01296640	
708601	LOC708601	similar to GULP,	Mmu.11298	XM_001105327	−2.15
		engulfment adaptor PTB		AANU01249499	
		domain containing 1		AANU01249498	
				XM_001105119	
				AANU01249495	
				XM_001105477	
				AANU01249497	
				AANU01249496	
				AANU01249507	
				AANU01249506	
				XM_001105193	
				AANU01249509	
				AANU01249508	
				AANU01249503	
				AANU01249502	
				AANU01249505	
				XM_001105407	
				AANU01249504	
				AANU01249510	
				AANU01249501	
				AANU01249500	
721477	OAZ1	ornithine decarboxylase	Mmu.3213	CO644742	−1.06
		antizyme 1		CB553280	
				NM_001134900	
				XM_001117645	
				CB310088	
				AANU01111056	
693317	PAIP2	poly(A) binding protein interacting protein 2	Mmu.2927	XM_001082025	−2.77
				XM_001082151	
707725	PDGFA	platelet-derived growth factor alpha polypeptide	#N/A	XM_001096150	−1.46
697772	PDK1	pyruvate dehydrogenase kinase, isozyme 1	Mmu.2590	XM_001086316	−1.52
706325	PGK1	phosphoglycerate kinase 1	Mmu.4126	XM_001100787	−1.46
				XM_001100332	
				XM_001100617	
				XM_001100701	
				DQ147960	
716665	PRDX2	peroxiredoxin 2	Mmu.2032	XM_001108992	−2.34
				XM_001109106	
				XM_001109159	
				XM_001109216	
				XM_001109048	
696171	SERPINH1	serpin peptidase inhibitor, clade H (heat shock protein 47), member 1, (collagen binding protein 1)	Mmu.3117	XM_001084827	−4.02
706593	SLC16A1	solute carrier family 16,	Mmu.10117	XM_001108968	−2.69
		member 1		DQ147927	
				XM_001109027	
				XM_001109083	
				XM_001109138	
				XM_001108877	
715915	SLC2A3	solute carrier family 2	Mmu.2873	XM_001113093	−3.13
		(facilitated glucose	Mmu.16589	XM_001113033	
		transporter), member 3		XM_001113127	
				XM_001113065	
				XM_001113218	
				XM_001112912	
				XM_001112821	
722154	SLC2A5	solute carrier family 2 (facilitated glucose/fructose transporter), member 5	Mmu.11703	XM_001118341	−1.4
719075	SLC25A1	solute carrier family 25 (mitochondrial carrier; citrate transporter), member 1	Mmu.10146	XM_001112697	1.59
574096	SOD1	superoxide dismutase 1,	Mmu.882	NM_001032804	−1.57
		soluble		AB087271	
704930	SPP1	secreted phosphoprotein 1	Mmu.225	XM_001093307	−2.9

The q-value is calculated as log2 fold change.

### Differential expression of oxygen-regulated and metabolic genes

Oxygen-regulated gene expression is known to be important for preimplantation embryo development [21]. The oxygen concentration in which the rhesus preimplantation embryo develops *in vivo* is reduced [53], [54] compared with *in vitro* culture. The HIF1A pathway was identified as over-represented in the significantly downregulated gene list by Bibliosphere, the 3881 significant gene list was further interrogated for HIF-regulated genes. Significantly, HIF1A transcript levels were 5.5 fold lower in *in vitro* ESC (q-value −2.462) than in *in vivo* ESC. In addition to the 18 genes identified in the HIF1A canonical pathway by Bibliosphere (**Table 2**), a further 17 genes known to be regulated by oxygen, including *SLC2A3* (glucose transporter 3), *ALDOA* (aldehyde dehydrogenase A) and *ENO1* (enolase 1), were identified in the 3881 differentially expressed gene list (**Table 5**). A comparison of the 3881 output with that of Rinaudo et al 2006 [55], examining the effect of oxygen on preimplantation mouse embryos, resulted in the identification of an additional 23 genes that appear to be regulated by oxygen during early development [55] (**Table 6**).

**Table 5:** Oxygen-regulated genes displaying differential expression between rhesus ESC generated from *in vivo* derived or *in vitro* cultured embryos compared with published data.

Gene Symbol	Gene Name	UniGene ID	Accession Number(s)	q-value
ADM	Adrenomedullin	Mmu.1495	XM_001100827	−2.23
			XM_001100373	
			XM_001100748	
AKT1	v-akt murine thymoma viral	Mmu.1599	XM_001085746	1.70
	oncogene homolog 1		XM_001085495	
			XM_001085265	
			XM_001085623	
			XM_001085152	
ALDOC	aldolase C, fructose-	Mmu.2882	XM_001107579	−1.10
	bisphosphate		XM_001107637	
BHLHE40	basic helix-loop-helix family, member e40	Mmu.2936	XM_001095506	−1.38
BNIP3L	BCL2/adenovirus E1B	Mmu.4295	NM_001037284	−1.15
	19 kDa interacting protein 3-		AY680445	
	like		CN641767	
CITED2	similar to Cbp/p300-	Mmu.12809	XM_001096152	−2.06
	interacting transactivator,		AANU01207265	
	with Glu/Asp-rich carboxy-terminal		AANU01207264	
COPS5	COP9 constitutive	Mmu.4188	XM_001097450	−2.30
	photomorphogenic homolog		XM_001097856	
	subunit 5 (Arabidopsis)		XM_001097650	
			XM_001097549	
			XM_001097759	
			XM_001098042	
CREB1	cAMP responsive element binding protein 1	Mmu.13784	XM_001107192	−1.48
CTGF	connective tissue growth factor	Mmu.3969	XM_001104316	−2.11
CTSD	cathepsin D	Mmu.2920	XM_001091374	−1.18
			XM_001091495	
			XM_001091601	
CXCL12	chemokine (C-X-C motif)	Mmu.3714	AF449283	−2.44
	ligand 12 (stromal cell-derived factor 1)		NM_001032934	
EDN1	endothelin 1	Mmu.13776	XM_001089874	−1.88
ENO1	enolase 1	Mmu.4213	XM_001098675	−1.13
			XM_001098378	
			XM_001098480	
			XM_001098286	
			XM_001098980	
			XM_001098778	
			XM_001098572	
			XM_001099088	
			XM_001097982	
			XM_001098883	
ETS1	v-ets erythroblastosis virus	Mmu.13289	XM_001113071	−1.32
	E26 oncogene homolog 1		XM_001113198	
	(avian)		XM_001113164	
			XM_001113134	
HIF1A	hypoxia inducible factor 1,	Mmu.4843	XM_001098939	−2.46
	alpha subunit (basic helix-		XM_001098836	
	loop-helix transcription		XM_001099043	
	factor)		XM_001098731	
			XM_001098338	
			XM_001099149	
			XM_001098630	
HMOX1	heme oxygenase (decycling) 1	Mmu.10024	XM_001113241	−1.56
HSP90B1	tumor rejection antigen	Mmu.1931	XM_001095189	−2.50
	(gp96) 1		DQ147987	
IGFBP2	insulin-like growth factor binding protein 2, 36 kDa	Mmu.10509	XM_00108707	−3.25
KRT18	similar to Keratin, type I	Mmu.7989	AANU01283678	−1.77
	cytoskeletal 18 (Cytokeratin-18) (CK-18) (Keratin-18) (K18)		XR_011513	
LGALS1	lectin, galactoside-binding,	Mmu.3924	EU152916	−2.28
	soluble, 1		XR_010795	
			NM_001168627	
LRP1	low density lipoprotein-related protein 1	Mmu.14648	XM_001099776	−1.19
MCL1	myeloid cell leukemia	Mmu.4052	XM_001102110	−1.99
	sequence 1 (BCL2-related)		XM_001102283	
			XM_001102191	
			XM_001101929	
MMP2	matrix metallopeptidase 2	Mmu.1027	XM_001087696	−1.50
	(gelatinase A, 72 kDa		XM_001087939	
	gelatinase, 72 kDa type IV		XM_001087814	
	collagenase)		XM_001087335	
NCOA2	nuclear receptor coactivator 2	Mmu.14283	XM_001082161	−1.04
PDGFA	platelet-derived growth factor alpha polypeptide	N/A	XM_001096150	−1.46
PDK1	pyruvate dehydrogenase kinase, isozyme 1	Mmu.2590	XM_001086316	−1.52
PGK1	phosphoglycerate kinase 1	Mmu.4126	XM_001100787	−1.46
			XM_001100332	
			XM_001100617	
			XM_001100701	
			DQ147960	
PKM2	pyruvate kinase, muscle	Mmu.9617	XM_001090817	−3.33
			XM_001090466	
			XM_001090930	
			XM_001091054	
			XM_001091297	
			XM_001091178	
			XM_001090238	
			XM_001090703	
			XM_001091427	
PPP5C	protein phosphatase 5,	Mmu.11271	XM_001111636	−1.79
	catalytic subunit		XM_001111674	
			XM_001111749	
			XM_001111714	
SLC2A3	solute carrier family 2	Mmu.2873	XM_001113093	−3.13
	(facilitated glucose	Mmu.16589	XM_001113033	
	transporter), member 3		XM_001113127	
			XM_001113065	
			XM_001113218	
			XM_001112912	
			XM_001112821	
SMAD2	SMAD family member 2	Mmu.2352	XM_001086377	1.50
			XM_001086616	
			XM_001086488	
SMAD3	SMAD family member 3	Mmu.14537	XM_001111078	−0.63
			XM_001111111	
			XM_001111262	
			XM_001111149	
			XM_001111187	
			XM_001111230	
SP1	Sp1 transcription factor	Mmu.3203	XM_001104877	−1.07
			XM_001104803	
			XM_001104948	
TFRC	transferrin receptor	Mmu.861	XM_001101412	−1.56
			XM_001101316	
			XM_001101222	
TXNIP	thioredoxin interacting	Mmu.3252	XM_001092636	−1.83
	protein		XM_001092517	
			XM_001092409	
VEGFA	vascular endothelial growth	Mmu.3550	AF339737	−1.14
	factor A		XM_001089925	
VIM	vimentin	Mmu.2647	XM_001093658	−2.22

The q-value is calculated as log2 fold change.

**Table 6:** Genes displaying differential expression between rhesus ESC generated from *in vivo* derived or *in vitro* cultured embryos and altered by oxygen in *in vitro* cultured preimplantation mouse embryos [55].

Gene Symbol	Gene Name	UniGene ID	Accession Number(s)	q-value
ARHGDIA	Rho GDP dissociation	Mmu.11137	XM_001112043	−1.29
	inhibitor (GDI) alpha		XM_001112147	
			XM_001112008	
CALR	calreticulin	Mmu.4315	XM_001110217	−1.92
			XM_001110174	
DHCR7	7-dehydrocholesterol	Mmu.15814	XM_001099101	−1.70
	reductase		XM_001099313	
			XM_001099202	
DHX9	DEAH (Asp-Glu-Ala-His)	Mmu.11214	XM_001114405	−2.75
	box polypeptide 9		XM_001114384	
GCDH	glutaryl-Coenzyme A	Mmu.15435	XM_001110430	1.340
	dehydrogenase		XM_001110384	
			XM_001110300	
GORASP2	golgi reassembly stacking	Mmu.1213	XM_001083589	−1.37
	protein 2, 55 kDa		XM_001083476	
			XM_001083797	
			XM_001083692	
HELLS	helicase, lymphoid-specific	Mmu.13556	XM_001094687	−2.97
			XM_001094310	
			XM_001095492	
			XM_001094077	
			XM_001095376	
			XM_001095601	
			XM_001094924	
			XM_001094189	
			XM_001095267	
			XM_001094806	
			XM_001095039	
			XM_001095698	
			XM_001095147	
HNRNPA2B1	heterogeneous nuclear	Mmu.2765	AANU01289359	−3.24
	ribonucleoprotein A2/B1		XM_001094282	
IDH1	isocitrate dehydrogenase 1 (NADP+), soluble	Mmu.2453	XM_001107875	−2.32
			XM_001107934	
			XM_001107627	
			XM_001107992	
			XM_001107810	
INPP5B	inositol polyphosphate-5-	Mmu.5966	AANU01008828	1.35
	phosphatase, 75 kDa		AANU01008826	
			AANU01008827	
			AANU01008824	
			AANU01008825	
			XR_013480	
			AANU01008823	
KIF22	kinesin family member 22	Mmu.14637	XM_001104522	−2.02
			XM_001104446	
			XM_001104204	
			XM_001104365	
			XM_001104124	
LOC694662	similar to Histone	Mmu.9710	XR_009889	−1.72
	deacetylase 2 (HD2)		AANU01296236	
			AANU01296235	
			AANU01296234	
			AANU01296233	
LOC695512	similar to RAB10, member	Mmu.9734	AANU01117583	−1.87
	RAS oncogene family		AANU01117585	
			AANU01117584	
			AANU01117587	
			AANU01117586	
			AANU01117595	
			AANU01117589	
			AANU01117594	
			AANU01117588	
			AANU01117593	
			XR_010252	
			AANU01117590	
			AANU01117591	
			AANU01117592	
LOC700557	similar to elongation of very	Mmu.14382	AANU01266409	−1.19
	long chain fatty acids		XM_001093537	
	(FEN1/Elo2, SUR4/Elo3,		XM_001093419	
	yeast)-like 1		XM_001093310	
LOC709018	similar to radixin	Mmu.12960	AANU01119660	−1.37
			AANU01119659	
			AANU01119658	
			XM_001104955	
			AANU01119657	
LOC711873	similar to eukaryotic	#N/A	AANU01107246	−1.69
	translation initiation factor		AANU01107245	
	2C, 2		XM_001100725	
LOC713958	similar to splicing factor,	Mmu.16625	XM_001103473	−1.72
	arginine/serine-rich 1		AANU01173069	
	(ASF/SF2)		AANU01173068	
			AANU01173071	
			AANU01173070	
			AANU01173072	
LOC714627	similar to basic leucine	Mmu.4082	AANU01288919	−2.01
	zipper and W2 domains 2		XM_001104484	
			AANU01288918	
			AANU01288921	
			AANU01288920	
LOC715977	similar to coactivator-	Mmu.4947	AANU01122653	−1.20
	associated arginine		AANU01122640	
	methyltransferase 1		AANU01122652	
			AANU01122642	
			AANU01122651	
			AANU01122641	
			AANU01122650	
			AANU01122644	
			AANU01122643	
			XR_013318	
			AANU01122646	
			AANU01122645	
			AANU01122647	
			AANU01122648	
			AANU01122649	
NDUFS4	NADH dehydrogenase	Mmu.2486	XM_001096222	−1.50
	(ubiquinone) Fe-S protein 4, 18 kDa (NADH-coenzyme Q reductase)		XM_001096347	
SCARB2	scavenger receptor class B,	Mmu.2325	XM_001096458	−1.25
	member 2		XM_001096341	
STK3	serine/threonine kinase 3 (STE20 homolog, yeast)	Mmu.976	XM_001095834	−1.22
UGP2	UDP-glucose	Mmu.466	XM_001085803	−1.25
	pyrophosphorylase 2		XM_001086473	
			XM_001086132	
			XM_001086361	
			XM_001086598	
			XM_001086015	

The q-value is calculated as log2 fold change.

In addition to perturbed expression of metabolic genes previously reported in preimplantation embryos, including *SLC2A1*, *SLC2A3*, *ALD2* and *PDK1*, regulatory genes controlling mitochondrial biogenesis were also identified as being downregulated in *in vitro* ESC, including *mtSSB*, *POLG* and *TFAM*, along with genes regulating mitochondrial dynamics (*MFN1*, *KIF5C* and *OPA1*; **Table S3**).

### Confirmation of gene expression by RT-PCR

To confirm the fidelity of our results, we assessed the expression of 13 genes identified in the data analyses. Genes involved in metabolism and mitochondrial function (*ATP5B*, *KIF5C*, *MFN1*, *PKM2*, *SLC2A3*, *UCP2*), pluripotency (*FGF2*, *POU5F1*, *SOX2*, *NANOG*), transcriptional repression (*PCGF2*), aging (*LMNA*) and embryo development (*FGF1R*, *IGF1R*, *IGFBP2*) were examined in pooled ESC RNA from available cultures (Ormes 7 and R466) grown under the same conditions as the samples used for transcriptional profiling. Expression of these genes was confirmed by RT-PCR, with all transcripts detected in both *in vitro* and *in vivo* ESC (**Figure S1**).

## Discussion

It is often overlooked that human ESC are generated from *in vitro* cultured, often surplus/‘discard’, embryos considered unsuitable for transfer in infertility clinics. While the classification of a good quality embryo is based largely on subjective criteria, it is well known that *in vitro* culture significantly perturbs embryo development, particularly in terms of gene expression, metabolism and subsequent development. With this in mind, we hypothesized that *in vitro* culture conditions would compromise gene expression in resulting ESC. To achieve this, we examined the transcriptional profiles of four different lines generated from *in vivo* derived embryos (R series) with that of four lines generated from *in vitro* derived embryos (Ormes series). Multiple passage numbers were analyzed to minimize passage related cell culture adaptation, with cells maintained under equivalent conditions known to support high quality ESC [56]. The data reported here represent selected passages between 8 and 37 for both *in vitro* and *in vivo* ESC. Transcriptional profiling of *in vitro* ESC and *in vivo* ESC identified a total of 3881 transcripts with twofold or greater differential expression, of which the majority were downregulated in *in vitro* ESC. Hierarchical clustering of ESC according to origin, irrespective of passage number, suggests that the differences in gene expression detected are stably maintained during long-term culture. It is important to consider that derivation of the R series (*in vivo*), and Ormes series (*in vitro*) carried out by different laboratories may contribute to some of the differences observed in the present study. However, as transcriptional profiles were compared over a range of early passage numbers, with all cell lines maintained under the same conditions by the same laboratory for each passage assessed, this contribution is likely to be minimal.

### *In vitro* ESC and *in vivo* ESC differ in the expression of imprinted and cell cycle genes, a potential legacy of embryo culture

Aberrant imprinting has been reported in a number of species following preimplantation embryo culture *in vitro* [57], [58], including the rhesus macaque [59], with long-term consequences for fetal growth and adult health [29], [33]. Bertolini et al [26] and Yaseen et al [60] have reported significantly decreased expression of *IGF1R* and *IGF2R* following *in vitro* culture of bovine embryos, conditions also associated with altered fetal and placental development and large offspring syndrome [27]. The expression of these genes was significantly lower in *in vitro* ESC when compared with *in vivo* ESC, suggesting that the altered expression of these genes in cultured embryos is preserved during ESC isolation. In support of this, a number of other genes involved in epigenetic regulation, including histones, histone deactylases and lysine-specific demethylase 3A were identified as differentially expressed between *in vitro* ESC and *in vivo* ESC (**Table S3**). Studies have also reported aberrations in imprinted genes in mouse [61], monkey [62], [63] and human ESC [64]–[67], particularly that of *IGF2* and *IGF2R*. Frost et al [68] reported genomic instability in human ESC, and suggested that derivation and ESC culture contributed to atypical methylation patterns, however it is possible that aberrant imprinting was inherent to the embryo from which the line was derived, in addition to any derivation and culture induced alterations. Significantly, epigenetic differences have been observed between mouse ESC generated from *in vitro* versus *in vivo* embryos [37], although these differences were lost by passage 5. Bioinformatic analysis of significantly different transcripts between *in vitro* and *in vivo* ESC also highlighted dysregulation of canonical pathways, particularly those regulating cyclins, cell cycle checkpoints and chromosomal stability (**Table 2**), including genes involved in the G1 to S phase known to be important in ESC [69], [70]. Mtango and Latham [71] have reported altered expression of cell cycle machinery in *in vitro* cultured rhesus embryos, suggesting that cell cycle control mechanisms may also be heritable from the embryo to resulting ESC. Misregulation of imprinted and cell cycle genes, previously documented following *in vitro* embryo culture, may therefore be preserved in resulting ESC, and may compromise the cells functionality during and/or following differentiation.

### *In vitro* culture perturbs the expression of key pluripotency regulators

Among the genes identified as significantly altered between ESC of different origin were known pluripotency markers, including *POU5F1* (*OCT4*), basic FGF and *SOX2*. Basic FGF (*FGF2*) is an important component of primate ESC culture media required for propagation and colony maintenance. FGFs play several roles *in vivo* during early development [72] and are known to mediate IGF expression [73], representing a positive feedback loop. Sato et al [6] reported that *FGF2* and *FGFR1* were important genes enriched in the undifferentiated state, regulated by *OCT4*, *SOX2* and *NANOG*. Activation of SMAD2/3 signaling is required for human ESC pluripotency [74] as both SMAD2/3 and FGF2 regulate *NANOG* gene expression. While *NANOG* is not significantly different between *in vivo* and *in vitro* ESC, *in vitro* ESC displayed significantly increased *SMAD2* expression. Upregulation of *SMAD2* may support ongoing culture in reduced levels of other pluripotency regulators. A reduction in the expression of *OCT4* and *SOX2*, in addition to a reduction in *FGF2* and FGF receptor expression, suggests that *in vitro* ESC may be more prone to spontaneous differentiation. Indeed, Byrne et al [55] reported significant variability in *OCT4* expression across the same Ormes lines examined in the present study. Less than a two-fold difference in the level of *OCT4* expression has been shown to have significant effects on ESC maintenance [75]. In support of this, Mtango et al [76] documented changes in pluripotency and differentiation marker expression during the early stages of rhesus macaque blastocyst outgrowth, and in Ormes 6 ESC, when compared with gene expression profiles of rhesus inner cell mass cells. Data therefore suggests that ESC derived from *in vitro* cultured embryos display alterations in pluripotency markers, however cells have potentially compensated by modulating other pathways to maintain self-renewal.

### The effects of oxygen on *in vitro* cultured embryos are sustained in ESCs

A significant difference between *in vivo* derived embryos and *in vitro* cultured embryos is the oxygen environment in which they develop. *In vivo* the oxygen concentration approximates 2–7% [52], [53], with an oxygen concentration of 2% reported in rhesus macaque uteri, considerably lower than the atmospheric conditions commonly used for *in vitro* embryo culture, and lower than the 5% oxygen concentration used to generate the embryos from which the *in vitro* ESC were derived. The oxygen environment is known to alter blastocyst gene expression and embryo development [21], [77]. Hypoxia-inducible factors (HIFs) are oxygen-sensitive transcription factors that mediate cellular adaptation to reduced oxygen conditions. HIF1 protein levels increase exponentially at oxygen concentrations lower than 6% [78]. The response to hypoxia leads to the activation of signaling pathways involved in the regulation of mitochondrial function, glycolytic metabolism and cell survival. In the present study, HIF1 alpha was significantly reduced in *in vitro* ESC (**Table 1**). Further analysis demonstrated enrichment (P = 0.0004) of HIF1 alpha regulated genes (**Table 5**). Physiological oxygen concentrations also regulate human ESC pluripotency, proliferation, karyotypic stability and differentiation [15], [79]–[82], mediated by HIFs [83]. Consistent with our findings, significant differences in *OCT4* levels [83], [84] and *SOX2* mRNA expression [83] have been reported in human ESC lines derived under 5% and 20% oxygen, or following transfer to reduced oxygen culture conditions. Significantly reduced expression of *FGFR1* and *FGFR2* [80] and *SLC2A3*, *PKM2*, *ALDOC*, and *LGALS1* [17] have also been reported in human ESC in response to atmospheric oxygen conditions, and differences in *SLC2A1*, *SLC2A3* and *PGK1* have been reported between in vivo derived and in vitro produced rhesus macaque blastocysts [85]. These results suggest that underlying alterations in metabolism may exist. This is further supported by downregulation of regulatory genes controlling mitochondrial biogenesis and dynamics in *in vitro* ESC, including *mtSSB*, *POLG* and *TFAM*, as well as *MFN1*, *KIF5C* and *OPA1* (**Table S3**). Differences in the expression of genes regulating mitochondrial biogenesis has also been reported between *in vivo* and *in vitro* rhesus blastocysts [86]. Significantly, Wale and Gardner [87] demonstrated that developmental perturbations observed following culture of preimplantation mouse embryo under atmospheric conditions were not restored by transferring cultures to a low oxygen environment, suggesting that adaptation of ESC will likewise not resolve underlying differences in ESC physiology. ESC properties may therefore be dependent on reduced oxygen conditions not only during derivation and subsequent expansion, but also during embryo culture prior to derivation.

## Conclusions

Results of the present study document significant differences at the transcriptional level between embryonic stem cells derived from *in vitro* cultured embryos, and those derived from *in vivo* derived embryos. Data suggests that embryonic stem cells may retain a transcriptional memory representative of the environment of the preimplantation embryo from which the cells were derived. *In vitro* ESC exhibit transcriptional perturbations seen in *in vitro* cultured embryos, including alterations in markers of pluripotency and differences impacted by oxygen concentration. These differences may impact cell physiology, although it is unclear whether these differences will contribute to long-term functionality following ESC differentiation and transplantation. Further investigation into the differences between *in vitro* and *in vivo* ESCs, particularly in terms of imprinting, metabolism and functionality following differentiation, is warranted to ensure their therapeutic potential. Attention needs to be directed towards physiological measures of functionality, coupled with transcriptional, epigenetic and proteomic characterizations of pluripotency, to assess the impact the culture environment has throughout stem cell isolation, maintenance and differentiation. As methods become more refined and more efficient, and xeno-free isolation becomes routine, the examination of not only embryonic stem cells, but also induced pluripotent stem cells will be pivotal in establishing fundamental properties necessary to supply normal, safe and efficient cells for therapeutic translation.

## Materials and Methods

### Embryonic Stem Cell culture

Four rhesus (*Macaca mulatta*) ESC lines generated from *in vitro* cultured embryos cultured up to day 9 (Ormes 6, 7, 10 and 13, [40]; referred to as ‘*in vitro* ESC’) and four lines generated from *in vivo* derived embryos flushed from uteri 6 days post ovulation (R-series 278, 366, 394 and 511, [41]; referred to as ‘*in vivo* ESC’) were cultured as previously described [56] and were generously provided by Dr Shoukhrat Mitalipov. Briefly, ESC were grown on mitotically inactivated mouse embryonic fibroblast feeder cells (MEF; cell line isolation was approved by the Oregon Health and Sciences University's Institutional Animal Care and Use Committee issued to S. Mitalipov) in Dulbecco's Modified Eagle Medium (DMEM/F12) (Invitrogen, Grand Island, NY) supplemented with 15% fetal bovine serum (FBS) (Hyclone, Logan, UT), 0.1 mM ß-mercaptoethanol, 1% nonessential amino acids (Invitrogen), 2 mM L-glutamine (Invitrogen), and 4 ng/ml FGF2 (Sigma), at 37°C under a 5% CO_2_-balance air atmosphere, and were passaged by manual scraping. To account for variability between derivation conditions, cultures were sampled from varying passage numbers (range 8–37) and cultures characterized to ensure that pluripotent ESC morphology, marker expression and karyotype were maintained.

### RNA extraction, microarray probe preparation and hybridisation

ESC colonies were collected following manual removal of MEFs and careful dissection to ensure no feeder cell transfer prior to lysis. Total RNA was isolated from cultures for each respective ESC line using TRIZOL reagent (Invitrogen), followed by further purification with a RNeasy MinElute Cleanup Kit (Qiagen). The RNA samples were quantified using a NanoDrop ND-1000 spectrophotometer (NanoDrop Technologies, Wilmington, DE) and the quality of the RNA was assessed using Lab-on-a-Chip RNA Pico Chips and a 2100 Bioanalyzer (Agilent Technologies, Palo Alto, CA). Samples with electropherograms showing a size distribution pattern predictive of acceptable microarray assay performance were considered to be of good quality. Twenty nanograms of total RNA from each line was amplified and labeled using a two-cycle cDNA synthesis and an *in vitro* transcription cRNA-RNA labeling system (GeneChip One-Cycle Target Labeling and Control Reagents; Affymetrix, Inc., Santa Clara, CA). Following successful cRNA amplification, 10 µg of labeled target cRNA was hybridized to Rhesus Macaque Genome Arrays (Affymetrix, Santa Clara, CA) using standard protocols, as described in the Affymetrix GeneChip Expression Analysis manual. Arrays were scanned using the GeneChip laser scanner (Affymetrix).

### Bioinformatic analysis

All microarray data complies with MIAME guidelines, and all microarray information and individual cell intensity (CEL) files are available online at the Gene Expression Omnibus (GEO; GSE25198). Analysis of Affymetrix output files was performed with DNA-Chip Analyzer (dChip; Harvard School of Public Health, Boston, MA) and Genomatix (www.genomatix.de) software. *In vivo* ESC samples were used as the baseline for comparison. For dChip analysis, data normalization and model expression was undertaken using default dChip settings, with analysis of the False Discovery Rate (FDR) also performed. A gene was defined as significantly up- or down-regulated if the signal fold-change between the target samples was greater than 2, at a significance level of alpha = 0.05. For Genomatix data analysis, statistical significance of differential gene expression was assessed by computing a q-value (logarithm) for each gene. Genes were considered to be up- or down-regulated when the logarithm of the gene expression ratio was more than 1 or less than -1, that is, a 2-fold or greater difference in expression, where alpha<0.05. Bibliosphere Pathway Edition (Genomatix), which combines literature analysis with genome annotation and promoter analysis, was used to create a directed regulatory network from transcripts identified by ChipInspector. To establish pathway and common framework information for significantly different transcripts, data was uploaded into GePS (www.genomatix.de). To further classify differentially expressed genes, Entrez gene IDs from the Genomatix analyses were used to search for over-represented biological processes against the rhesus and human genomes. Gene Ontology was performed using NetAffx (www.affymetrix.com).

### RT- PCR validation

To validate the microarray results, RT-PCR was carried out on representative rhesus ESC samples (Ormes 7 *in vitro* and R475 *in vivo*) for 13 genes identified as significantly altered by the microarray analyses. RNA was extracted using an Absolutely RNA Nanoprep Kit (Stratagene, La Jolla, CA, USA), from which 1 µg was reverse transcribed into cDNA using SuperScript III reverse transcriptase (Invitrogen) and random primers (Applied Biosystems, Foster City, CA, USA) according to the manufacturer's instructions. Resulting cDNA was amplified with 1U Taq polymerase (Qiagen, Valencia, CA) in a final volume of 50 µl containing 1× buffer, 1.5 mM MgCl2, 10 pmol of each sequence-specific primer and 10 mM of each dNTP. The mixture was amplified for 40 cycles in a BioRad DNA Engine thermal cycler (BioRad, Hercules, CA), where each cycle included denaturation at 94°C for 1 min, reannealing for 30 sec at 60°C, and primer extension at 72°C for 30 sec, followed by a final extension at 72°C for 7 min. PCR products were analyzed by electrophoresis through 2% agarose gels containing 0.5 mg/ml ethidium bromide and were photographed using a Kodak GL100 Imaging System equipped with Kodak Molecular Imaging software (Eastman Kodak Co., Rochester, NY). Primers were designed using Primer Express software (Applied Biosystems, Foster City, CA) and are listed in **Table S1**.

## Supporting Information

Figure S1 RT-PCR analysis of undifferentiated rhesus ESC generated from in vitro (A) or in vivo (B) derived embryos.
(TIF)

Table S1 PCR primer sequences used for validation of microarray results.
(DOCX)

Table S2 dChip output generated from CEL files (GEO: GSE25198; http://www.ncbi.nlm.nih.gov/geo/query/acc.cgi?acc=GSE25198).
(XLSX)

Table S3 Genomatix output generated from CEL files (GEO: GSE25198; http://www.ncbi.nlm.nih.gov/geo/query/acc.cgi?acc=GSE25198).
(XLSX)

Table S4 Transcripts identified within common frameworks CTCF-HIFF, ETSF-HIFF and SMAD-E2FF.
(XLSX)
