# Supplementary figures and images for: Transcriptional Differences between Rhesus Embryonic Stem Cells Generated from In Vitro and In Vivo Derived Embryos
Source: PLoS One. 2012 Sep 18;7(9):e43239. doi: 10.1371/journal.pone.0043239 (PMC3445581; doi:10.1371/journal.pone.0043239)

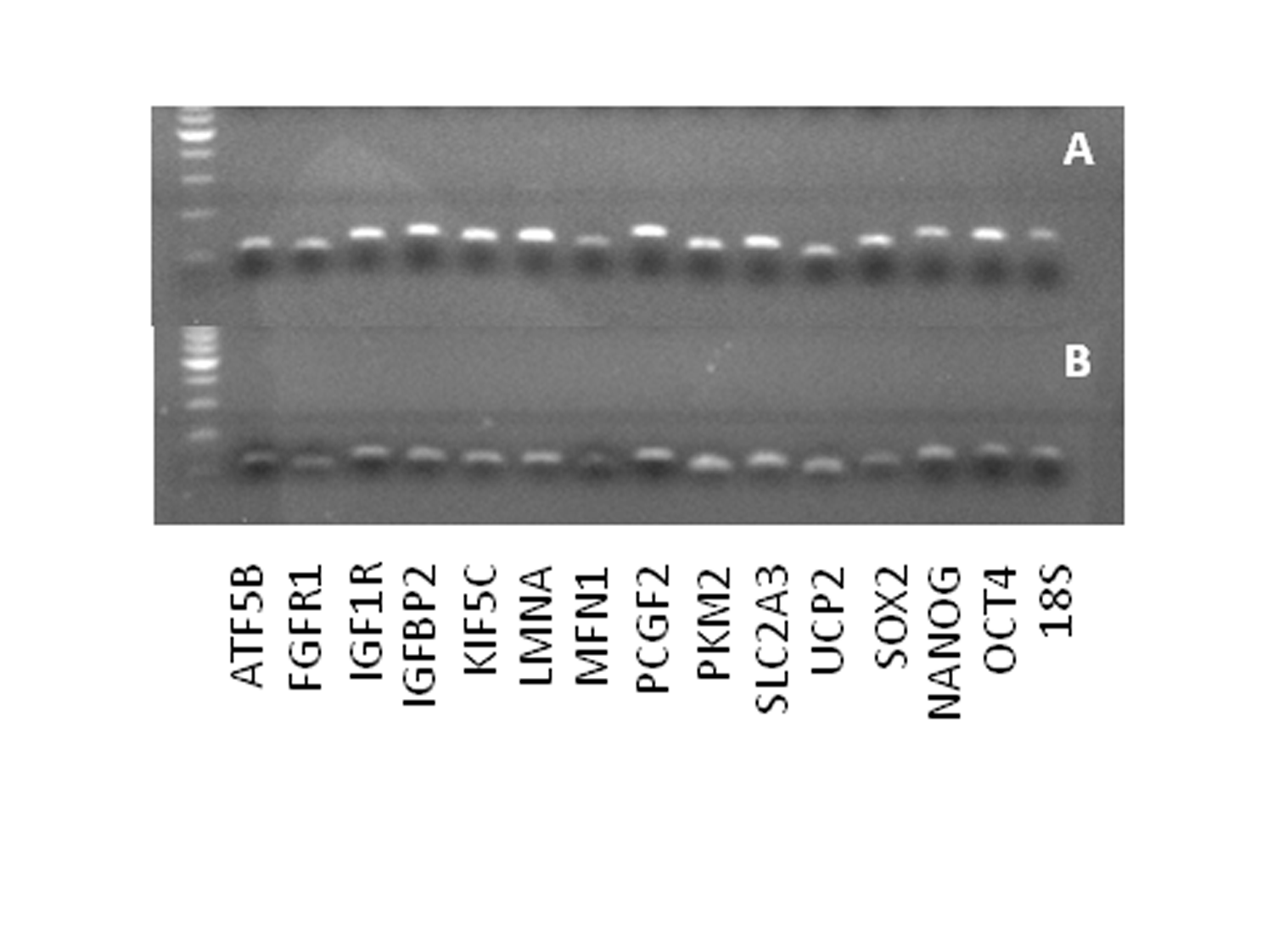

Supplement: Figure S1 — RT-PCR analysis of undifferentiated rhesus ESC generated from in vitro (A) or in vivo (B) derived embryos. (TIF) [file pone.0043239.s001.tif]
