# Supplementary material for: Transcriptional Differences between Rhesus Embryonic Stem Cells Generated from In Vitro and In Vivo Derived Embryos
Source: PLoS One. 2012 Sep 18;7(9):e43239. doi: 10.1371/journal.pone.0043239 (PMC3445581; doi:10.1371/journal.pone.0043239)
Supplement: Table S1 — PCR primer sequences used for validation of microarray results. (DOCX) [file pone.0043239.s002.docx]

**Suppl Table 5.** Primer sequences for RT-PCR validation of microarray results.

| Gene | Primer | Primer Sequence |
| --- | --- | --- |
| 18S | 5’ | AGAAACGGCTACCACATCCAA |
|  | 3’ | CCTGTATTGTTATTTTTCGTCACTACCT |
| ATP5B | 5' | AGCGCTGGTATATGGTCAAATGA |
|  | 3' | CCTGGGTGAAGCGAAAGATATTAT |
| FGF2 | 5' | GACCCCAAGCGGCTGTACT |
|  | 3' | ACTCCTTTGATAGACACAACTCCTCTCT |
| FGF1R | 5' | TCTGCGTGGCTCACTGTCA |
|  | 3' | ACCCTACCATGCAGGAGATGA |
| IGF1R | 5' | CCTCAGGACGGCTACCTTTACC |
|  | 3' | ACACCTCAGTCTTCGGGTTCTCT |
| IGFBP2 | 5' | ATGCGCCTTCCGGATGA |
|  | 3' | CACGCTGCCCGTTCAGA |
| KIF5C | 5' | CAAACATGAATGAACACAGCTCTAGAA |
|  | 3' | CTCCCAGCCAAATCAACCAA |
| LMNA | 5' | CGCAAGACCCTCGACTCAGT |
|  | 3' | GCCATCAGGTCACCCTCCTT |
| MFN1 | 5' | CATGCCCTTCACATGGACAA |
|  | 3' | TGGACTGTCTACTAACACCAGGTCAT |
| Nanog | 5’ | ATTCAGGACAGCCCTGATTCTTC |
|  | 3’ | TCTGTTTCTTGACCGGAACCTT |
| POU5F1 | 5’ | CCAGTATCGAGAACCGAGTGAGA |
|  | 3’ | GAACCACACTCGGACCACATC |
| PCGF2 | 5' | CGGACTACACGGATCAAAATCAC |
|  | 3' | CAGGTAGCGCACGATGCA |
| PKM2 | 5' | ACCACTTGCAATTATTTGAGGAACT |
|  | 3' | CCACTGCAGCACTTGAAGGA |
| SLC2A3 | 5' | GTCCTTGTCTGTGGCCATATTCTC |
|  | 3' | CAGCGACAGCCAACAGGTT |
| SOX2 | 5’ | GAGAACCCCAAGATGCACAACT |
|  | 3’ | CGCTTAGCCTCGTCGATGA |
| UCP2 | 5' | GCTAAAGTCCGGTTACAGATCCA |
|  | 3' | CGGCAACCAGTCCATTGTAGA |
